# A Novel Human Activity Recognition and Prediction in Smart Home Based on Interaction

**DOI:** 10.3390/s19204474

**Published:** 2019-10-15

**Authors:** Yegang Du, Yuto Lim, Yasuo Tan

**Affiliations:** School of Information Science, Japan Advanced Institute of Science and Technology, 1-1 Asahidai, Nomi, Ishikawa 923-1211, Japan; ylim@jaist.ac.jp (Y.L.); ytan@jaist.ac.jp (Y.T.)

**Keywords:** human activity recognition, object usage sensing, activity prediction, RFID, smart home

## Abstract

Smart Homes are generally considered the final solution for living problem, especially for the health care of the elderly and disabled, power saving, etc. Human activity recognition in smart homes is the key to achieving home automation, which enables the smart services to automatically run according to the human mind. Recent research has made a lot of progress in this field; however, most of them can only recognize default activities, which is probably not needed by smart homes services. In addition, low scalability makes such research infeasible to be used outside the laboratory. In this study, we unwrap this issue and propose a novel framework to not only recognize human activity but also predict it. The framework contains three stages: recognition after the activity, recognition in progress, and activity prediction in advance. Furthermore, using passive RFID tags, the hardware cost of our framework is sufficiently low to popularize the framework. In addition, the experimental result demonstrates that our framework can realize good performance in both activity recognition and prediction with high scalability.

## 1. Introduction

Over the last few years, the Internet of Things (IoT) has been greatly developed with the help of mobile computing, edge computing, and cloud computing. One of the most representative applications of IoT is smart home, which has good prospects in the future. In a typical smart home system, all the resources and devices can be controlled by the smart home platform. The long-term goal of smart home is to achieve automatic control according to both environment and inhabitant. Owing to the advances in sensing technology, it is not difficult to obtain the environment data such as illumination, temperature, and humidity. However, still no practical solution exists to recognize human activity at home to enable scenario-based smart services [1]. To enable the smart home platforms to know more about their host, human activity recognition (HAR) has become an urgent challenge for the researchers.

Unlike the online consumer activity, the activity of daily living (ADL) usually cannot produce any data for a computer system, thereby causing a gap between inhabitant and smart home system. To bridge this gap, the existing work has shown us a bright direction. The interaction between human and devices could be the channel to recognize the human activity. Wearable devices have already been widely used in recent years. Such devices equipped with different kinds of sensors and microprocessors are worn on the human body to monitor the state of humans [2]. Represented by Apple Watch, smart watches and smart wristbands can recognize simple activities such as waving hands and sitting still [3,4,5]. However, such activities do not contain any semantic meaning, and their recognition can be more appropriately called either gesture recognition or action recognition [6]. In this study, we define these kinds of activities as low-level activity and its detail will be covered in Section 2. These activities cannot be used directly by the smart home system to provide scenario-based services. Another way to detect the interaction between human and devices is to attach sensors to objects used extensively by humans [7]. However, smart electronic sensors rely on batteries, thus the size of these smart sensors is not sufficiently small to be attached to all the devices, not to mention their price along with their maintenance cost. It is worth noting that passive radio frequency identification (RFID) tags seem to have the ability to replace such sensors. In addition, several works have been proposed to prove that passive RFID tags offer a good way to detect object usage [8,9]. Based on these research works, our work achieves even further results of object–usage-based activity recognition.

**Figure 1:**
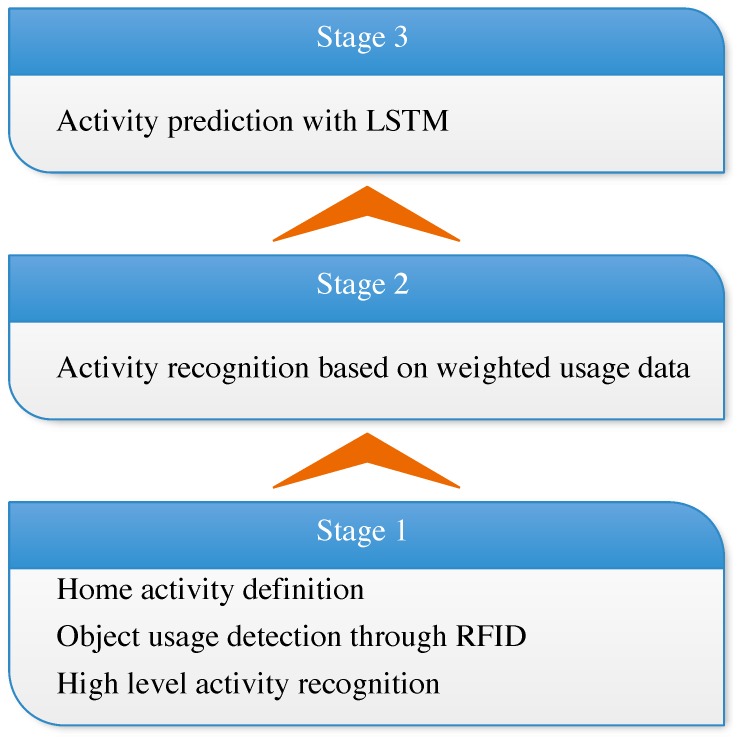
Three-stage framework to recognize and predict human activity in a smart home.

In this study, we first deeply analyze the characteristics of human activity in home environments. This further clarified the goal of HAR in smart homes; the goal is to provide scenario knowledge to the smart home platform to achieve human-centered automatic service. Then, we propose an RF-ARP framework recognize and predict the human activity in smart homes, as depicted in Figure 1. Different from address-resolution protocol (ARP), which translates an IP address to MAC address, our RF-ARP translates a wireless radio frequency (RF) signal to the human activity. The proposed RF-ARP framework mainly contains three stages: recognition after the human activity, recognition in progress, and activity prediction in advance. In the first stage, we utilize passive RFID tags to detect the interaction between human and device and recognize a high-level activity by combining those low-level activities. In this stage, we can record the activities of the inhabitants. Furthermore, in the second stage, we weight the device based on term frequency–inverse document frequency (tf-idf) to ensure the significance of each device to each activity. In this way, we will not have to give the recognition result after the completion of the activity—while, in the third stage, we already have the activity log. Thus, we could use the long short-term memory (LSTM) network to model the ADL of the inhabitant. In this manner, the proposed framework will be able to predict the next activity that, perhaps, happens after the current activity. We finally test our framework using off-the-shelf equipment and an open-source database, followed by proving the effectiveness and efficiency of RF-ARP.

Building such a system involves several challenges. The first one is to achieve object detection without using wearable devices. The existing work usually uses a wearable RFID reader to detect the object usage according to the distance between the object and the hand of the human [8]—while, in our work, we use a fixed long-distance antenna to cover as much region as possible, leading to the invalidation of the previously reported method. Therefore, we propose a way to detect object usage by phase, which is a physical feature of the RFID signal. The second challenge is the recognition of concurrent activities. We introduce a task-oriented generative approach rather than a discriminative approach; therefore, the recognized result could be more than one activity. In addition, traditional machine learning methods rely on training data, causing the so-called “cold start” problem. However, our approach utilizes the prior knowledge to define an activity, thus the training data are not required in the first stage. Thus, the upper stages could be in motion after sufficient data have been produced in the first stage.

Compared with the existing work on activity recognition in the smart home, our framework has multiple advantages regarding different aspects. First, scalability is the most important strength of our approach, which is reflected in two aspects. The first aspect is that we allow the smart home platform to define all kinds of activities as it needs. This is great progress, as the activity recognized by the existing work may be not what the smart home platform requires. In addition, the sort of activities could not be changed in the previously conducted work on HAR. The other aspect is that our approach could work in different houses, even when the objects and devices are different from each other. However, the existing work cannot migrate the trained model to adjust in a new environment, limiting the model to only work in the laboratory. In addition, our activity prediction stage is going further than the current HAR in smart homes. This may largely promote fully automatic smart homes in the near future. The next strength is that our three-stage framework unwraps the task to recognize the high-level activity from wireless signal data. This brings huge flexibility because every stage can be optimized independently or even replaced by other algorithms. For example, we can substitute the LSTM model in the third stage with any other time-series data-mining algorithm because the labeled data provided by the first stage can be used to train different models. The last strength of our framework is that both the cost of RFID tags and the computational complexity are sufficiently low to implement our framework in the current houses without much effort.

## 2. Preliminaries

Before introducing the HAR framework, the characteristic of activity and the goal of HAR in the smart home should be clarified. In this section, we deeply analyze the human activity and expound the effect that HAR should have in smart home systems.

To recognize a human activity, we have to know the habit of the human at home. Here, we explore the features of human activity at home as follows, and they may be the reasons why it is difficult to build a general HAR approach.

**Figure 2:**
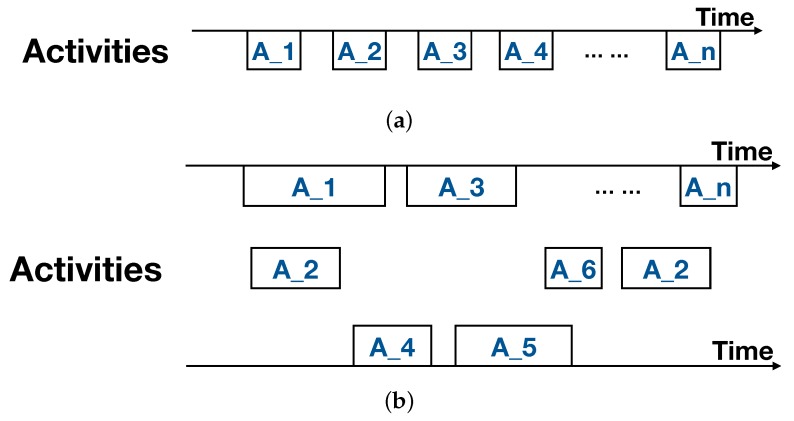
Comparison between the existing human activity recognition (HAR) approaches and the ground truth in the real world. (**a**) Result of the existing HAR approaches; (**b**) Activities in the real world.

- **Concurrency.** This is the biggest gap between the result of the HAR approach and the ground truth in the real world. A human usually performs different activities concurrently. As depicted in Figure 2b, activities A_1 and A_2 are simultaneously in progress—while the existing HAR approaches are basically discriminative, meaning they can only recognize one result at a moment, as depicted in Figure 2a.
- **Multiplicity.** It means that the sort of activities could be different as seen from different granularity. For example, the activity of “cooking” may involve subactivities such as “washing vegetables”, “cutting ingredients”, “frying” and so on. It is difficult to recognize a full-grain-size activity, as the HAR approach does not even know what kind of activity the smart-home platform needs to know. In this study, we grade the activities according to the semantic meaning they have. A high-level activity has more semantic meaning than that of lower ones [10].
- **Complexity.** An object or device could be used in different activities, and an activity may also use several devices. This means it is not impossible to infer the activity directly from object usage.
- **Diversity.** Owing to the difference in cultural, race, region, and even character, individuals may perform the same activity in completely different ways. This difference causes huge trouble to the training-based approach, as the trained model in the laboratory could not be applied to real houses.
- **Randomness.** Although the individuals have specific behavioral habits, it is not easy to excavate such patterns. This is because large randomness in activities exists in daily living, and because the next activity is decided not only by the current activity, but also by the earlier ones.

The above-mentioned five features render the existing research works impractical in smart homes. However, our work will overcome the challenges posed by these features and achieve the previously mentioned goals via the three-stage framework introduced in Section 3.

To provide knowledge to the smart home platform for realizing automatic control, an HAR approach should have the following abilities: (a) it should give the start time and the end time of each activity; (b) it should recognize full-grain-size activities; (c) it should be able to deal with concurrent activities; and (d) it should be scalable for different houses.

## 3. Design of the RF-ARP Framework

In this section, we introduce our framework in detail and show the capacity to achieve the goals mentioned in Section 2. The basic idea is to combine the devices with both the inherent semantic meaning and the human–device interaction, in order to detect the object usage. Furthermore, we infer higher level activities according to the definition of each activity. Using the log of recognized activities, we weight the devices by using tf-idf to recognize the activity while it is in progress. Finally, we utilize the LSTM to model the habit of ADL, following which we predict the activity that the inhabitant might perform later.

### 3.1. Object Usage Detection

In this section, we present a way to detect the object usage by using passive RFID tags and transform the physical-signal data to binary-state data.

Passive RFID tags are small and sufficiently cheap to be widely used in applications such as logistics and warehousing [11,12]. Moreover, researchers have found that RFID tags can be used to detect the object usage by attaching them on objects used every day [13]. Fazzzinga et al. even utilized RFID tags to trace the objects both online and offline [14,15,16]. This may considerably help in object–usage analysis. Several years before, Philipose et al. used a glove equipped with a near-field RFID reader to do such work by the state of readable and not of the tags [17]—while, in our work, we selected a long-distance antenna and ultra high frequency (UHF) reader to perform the same task. The reason is that a fixed long-distance antenna can cover a large area and scan all the tags in the area almost simultaneously. Furthermore, some objects are not used by hand, such as chairs and beds. In addition, we do not require the inhabitant to wear any devices, thereby reducing the inconvenience to the inhabitant.

Several systems have been proposed for achieving object–usage detection via a UHF RFID reader [18,19]. In these systems, the received-signal-strength indicator was merely used to distinguish the state of the tags; note that state is not a stable parameter. Our previous research explained that the RF phase of RFID tags can better reflect both the spatial attribute and the interaction between human and tagged objects [20].

**Figure 3:**
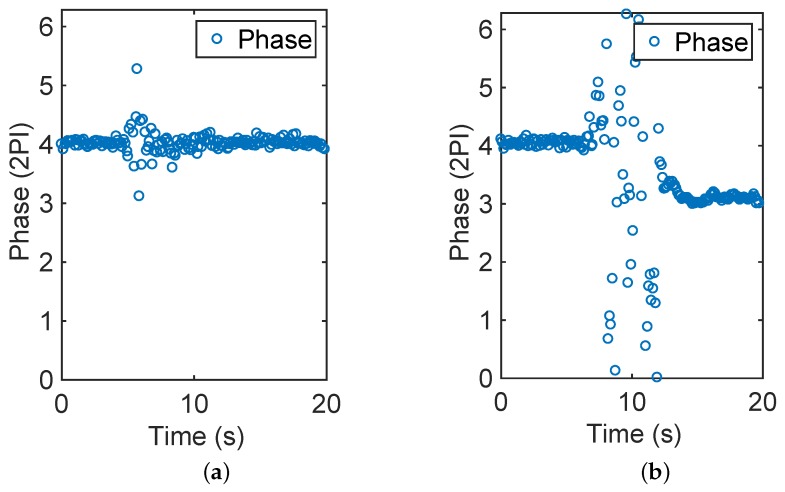
Interactions and the corresponding phase changes. (**a**) Passing by; (**b**) Picking up.

Phase is a back-scattered RF-channel parameter, which can be continuously read by a UHF RFID reader. In our work, we use Impinj R420 (Seattle, USA) as the reader to obtain the phase value. According to our previous research, phase is sensitive to the interaction between human and RFID tags. As depicted in Figure 3, different interactions change the phase value in varying degrees. Figure 3a depicts the condition in which there is a human passing by the object with an RFID tag—while, in Figure 3b, the human walks to the object and picks it up, then puts it back to its position and walks away. This inspires us to use the dispersion of the phase data in a sliding window to distinguish among the interactions. We have then proved that only if the object is picked up will the tag state be revised as “1”, irrespective of how close the people are standing or passing by the object. Because phase is more sensitive to distance than interferences or reflections, even when several people are close to one another, their interactions with the objects could be detected correctly. The detailed analysis of this phenomenon is proposed in our previous work [20].

Apart from the above-mentioned interactions, people also interact with heavy furniture such as a bed and sofa without moving them. In spite of this, in these cases, the object usage can be detected by the phase. UHF RFID readers are able to scan the tags several times in a second. In our verification experiment, the average sampling rate for each tag is 12 times per second. Although the reader can keep receiving the back-scattered signal when the tag is interfered, it cannot see the tag while the tag is completely blocked. This enables us to use the following simple way to detect such interactions:

(1) $$covered=if(\left(t_{0}-t_{1}\right)>T),$$

where $t_{0}$ represents the current timestamp, $t_{1}$ represents the timestamp of previous round scanning, and *T* is the threshold of the tag state. In this study, we set *T* as 1 s to ensure enough sufficient sensitivity to detect short-term interactions. For some specific objects such as bed, we can increase *T* to detect the right interaction. Note that, when the tag works as a switch, one more step is required to translate the interaction to the equip state. The relevant details were introduced in our previous work [21].

**Figure 4:**
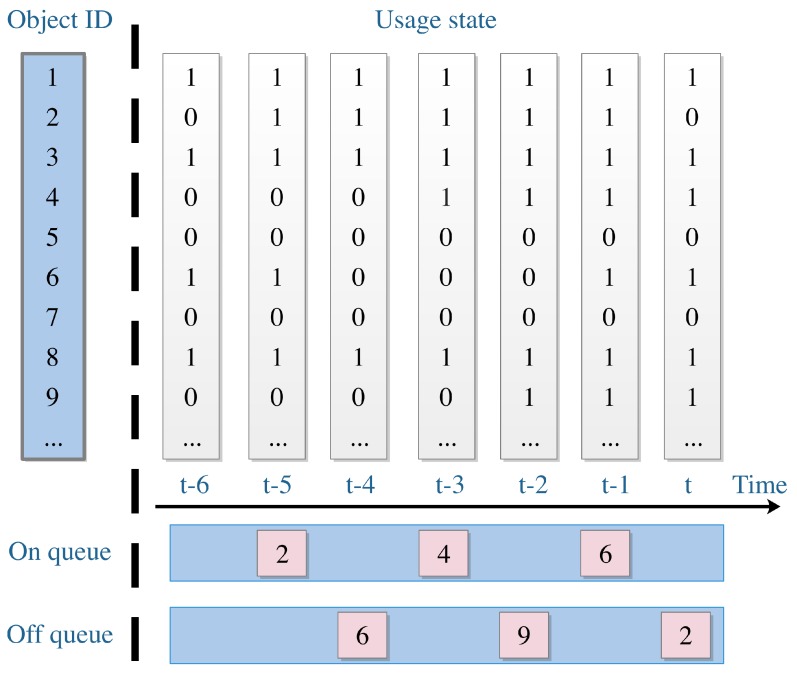
Object-usage state vector changes with time, and we can generate “On queue” and “Off queue”, respectively.

Thus far, we have introduced the way to detect object usage, and Table 1 presents the way to determine the object–usage state. When the tagged object is covered or picked up, it means the object is being used, and thus the usage state is set to “1”. Otherwise, when the tagged object is interfered or still, it means the object is not being used; thus, the usage state is set to “0”. As depicted in Figure 4, the matrix with a white background color is an example of object–usage array.

### 3.2. High Level Activity Recognition

In this section, we present a flexible and scalable approach to recognize high-level activities. The activity is recognized using a definition based on prior knowledge.

To ensure that the result of HAR is required by a smart home platform, the best solution is to grant the smart home platform the authority to define what it needs to know. Thus, we make a rule to enable such authority. The definition of an activity includes only the objects used in that activity. For example, television has a semantic meaning that greatly indicates the activity of “watching TV”.

As depicted in Figure 4, after detecting the usage states of the objects, we built two queues to store the detected usage states. The “On queue” contains the object ID and the timestamp that the object starts using—while the “Off queue” contains the object ID and the timestamp that the object stops using. At this stage, the length of the queues is fixed as one day. It means that the task of this stage is to make a record of activities performed during the last one day.

We now consider “watching TV” as an example, where $O_{2}$ represents the television. At t−5, the television is turned on, and it is turned off at *t*. Therefore, we can say that the activity of “watching TV” has been performed; in addition, the start time is t−5 and the end time is *t*. However, this is a merely single-object activity. In general, a high-level activity tends to include more than one object. Therefore, we set the start time as when the first object is used, and the end time when the last object stops being used. However, we cannot obtain the correct end time because the activity may be interrupted by other activities. In this case, we are confused regarding the time at which the activity ends.

**Figure 5:**
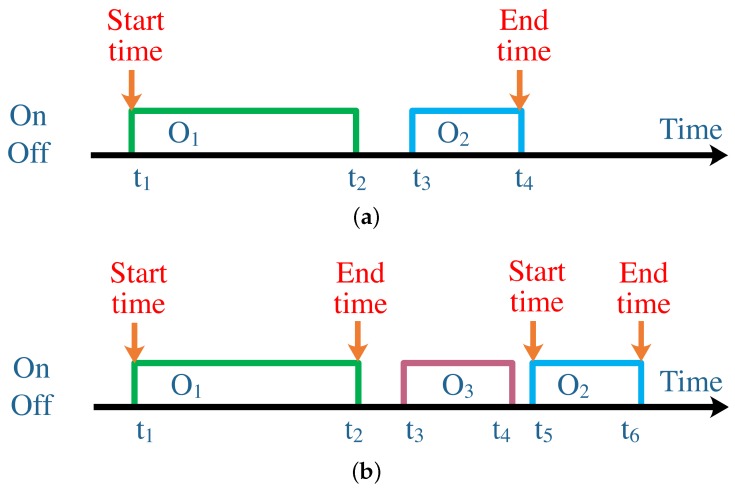
We propose two strategies to determine the start time and end time. $O_{1}$ and $O_{2}$ are objects that belong to one activity, and $O_{3}$ belongs to some other activity. (**a**) No interruption between two objects; (**b**) Interruption between two objects.

To overcome the aforementioned problem, we treat the object in the definition individually as a subset of the set that contains all the objects. In addition, two strategies are proposed to determine the start and end times, as depicted in Figure 5. In Figure 5a, there is no “On” action between $t_{1}$ and $t_{3}$, meaning that the activity is ongoing continuously, although the usage of $O_{1}$ ends before the usage of $O_{2}$ starts. Therefore, we can merge the two subsets to a new subset. The start time is still the timestamp of the first object in the “On queue”, and the end time is set as the timestamp of the last object in the “Off queue”. When the next object belonging to the same activity starts to be used, we check the interruption in the same way as above. If there is still no interruption, then we keep merging the subset to the former subset and reset the end time. However, when there is an interruption, we use the strategy depicted in Figure 5b. We can see that, between $t_{1}$ and $t_{5}$, $O_{3}$ has been used at $t_{3}$. Thus, in this case, the activity is interrupted by another activity. We, therefore, cut the relationship between the current subset and the former one that belongs to the same activity. The former subset has finished at $t_{2}$, so the end time is set to $t_{2}$. Moreover, the current subset becomes the initial subset, and the start time is $t_{5}$. However, the end time is $t_{6}$ and may be rewritten by the later subset too.

The reason why we chose these two strategies is because we wanted to recognize the activity as accurately as possible and reflect the real scene. Suppose, while cooking in the kitchen, that the inhabitant hears a phone call. After receiving the phone call, he resumes cooking. The former HAR approach can find it difficult to distinguish such a short-term activity from a continuous activity—while our strategies could record the ground truth in detail. Moreover, we allow each activity to create its own object set independently, so that the processes of HAR are also independent, even if an object may be involved in several processes. In this way, the multiple concurrent activities can all be recorded simultaneously.

### 3.3. Recognition in Progress

In the last section, we recognize the activity in an offline manner—while, in this section, we introduce an online approach that recognizes the activity in progress.

In the first stage of our framework, we define an activity by using the objects that are only used in that activity. Although it can record the log of activities that have been performed efficiently, the precision of the start and end times is not so satisfactory. In addition, the definition of activity has limitations. Many objects can not be included in the definition because they might be used in more than one activity. Thus, we introduce an approach to extend the definition of an activity, and, subsequently, recognize the activity based on the new definition.

In previously conducted research works, tf-idf was commonly used in both natural language processing and information retrieval [22,23]. It is a numerical statistic that is intended to reflect how important a word is to a document in a collection or corpus [24]. Owing to the ability to reflect the importance of element in frequency, we utilize the tf-idf to weight the objects according to the definition of activities. Before calculating the tf-idf value of each object, we have to generate the training data. In the first stage, the interval between two activities is large because of the very strict activity definition. Therefore, we extend the start time of one activity to the end time of the former one and extend the end time of the activity to the start time of the last one. The objects used to exist between two activities are involved in the definitions of the activities on both the sides of the usage. In this way, the definition of an activity is extended considerably.

Without loss of generality, we represent the set of objects *O* as

$$O=\{o_{1},o_{2},\ldots ,o_{n}\},$$

where *n* denotes the number of objects. The set of activities *A* is denoted as

$$A=\{a_{1},a_{2},\ldots ,a_{m}\},$$

where *m* denotes the number of activities. In addition, we define the set of activities in the process as P⊆A.

In the activity log data, we count the frequency of every objects $o_{i}$ (i∈n) that has been used in a specific activity $a_{j}$ and note it as $g_{i}^{j}$. Note that that our only concern is whether the object has been used in one round of the activity, ignoring the number of times it has been used. If the object is used more than once in one round of activity, we still count one for one round. Moreover, to reduce the noise caused by the activity–definition extension, we use the follow equation as a high-pass filter:

(2) $$g_{i}^{j}=\left\{\begin{array}{cc} 0, & g_{i}^{j}<z*max\left(g_{i}^{j}\right),\\ g_{i}^{j}, & otherwise, \end{array}\right.$$

where *z* denotes a threshold set as 0.5 to control the filter in our work. The higher we set the value of *z*, the stricter the definition of an activity becomes. Particularly, if we set *z* as 1, the activity definition will be the same as that in the first stage.

The term frequency $tf_{i}^{j}$ can be calculated using the follow equation:

(3) $$tf_{i}^{j}=\frac{g_{i}^{j}}{\sum_{i=1}^{n}g_{i}^{j}}.$$

Moreover, the inverse document frequency $idf_{i}^{j}$ can be calculated using Equations (4) and (5)

(4) $$idf_{i}^{j}=log\left(\frac{m}{\sum_{j=1}^{m}f_{i,j}\left(T\right)}\right),$$

(5) $$f_{i,j}\left(T\right)=\left\{\begin{array}{cc} 0, & g_{i}^{j}=0,\\ 1, & otherwise. \end{array}\right.$$

After obtaining $tf_{i}^{j}$ and $idf_{i}^{j}$, the tf-$idf_{i}^{j}$ then can be calculated as follows:

(6) $$tf-idf_{i}^{j}=tf_{i}^{j}*idf_{i}^{j}.$$

Using the training data, we can finally generate a weight matrix that illustrates the importance of each object to different activities. Then, we utilize this matrix to realize online activity recognition.

Similar to the first stage, when a new object usage is detected, the object ID *i* (i∈n) is put into the “On queue”. Subsequently, we start to check the weight matrix to obtain the maximum $tf-idf_{i}^{j}$ and the corresponding *j*. In other words, the weight matrix tells the most possible activity, as the object is most representative to that activity. Then, we need to check the set of activities in the process *P* to verify whether this activity is in set *P* or not. If it is in set *P*, we do not need to change anything and, thus, we keep waiting until the next object–usage–state change. However, if the activity is not in set *P*, we then add the activity ID in *P* and note the start time of this activity with the current timestamp.

When the usage of a new object is detected to have ended, we also need to check the weight matrix with the object ID *i*. We retrieve the activities whose $tf-idf_{i}^{j}$ are not 0, and then we withdraw all the relevant activities from the set *P* and set their end times, respectively. In addition, if the next activity is a part of the activities that have just been withdrawn, we merge them together as the first stage does, and then we set their end time to empty.

Note that the log of recognized activities is essential to determine the weight matrix. Moreover, to ensure the effectiveness of the proposed approach, the log data should be as large as possible. Furthermore, when a new activity or new device is involved, the weight matrix can to be updated automatically.

### 3.4. Activity Prediction Using LSTM

In the last section, we introduced the way to recognize the activity in real time. However, in this section, we will go further to predict the activity that may happen later. In the second stage, we obtain the log of activity with the weight matrix. In addition, owing to our manner of segmenting the activities, some activities may end simultaneously. Note that, for a real-world situation, several activities may be going on at the same time. However, we believe that those activities will not start together. Therefore, we utilize the start times of the recognized activities as a sequence to represent the order of activities.

In this research, we treat human activity prediction as a time sequence prediction problem. We believe that the inhabitants perform different activities in a relatively fixed pattern. For example, according to the activity log, there is an inhabitant who always watches TV after having dinner. Therefore, if the inhabitant is detected to be having dinner currently, then his/her next activity is most probably watching TV. Such a problem with predicting next state based on the current state can be solved using the classical machine learning approach. Nevertheless, the next activity is related with not only the current activity but also the previous ones. Therefore, we introduce deep learning to solve this problem. The recurrent neural network (RNN) performs well on spatial temporal predicting problems, such as location prediction [25]. LSTM networks are a special kind of RNN, and they are proved to be more efficient than RNN [26]. LSTM networks can memorize both long and short-term knowledge, thereby tallying with the human mind.

**Figure 6:**
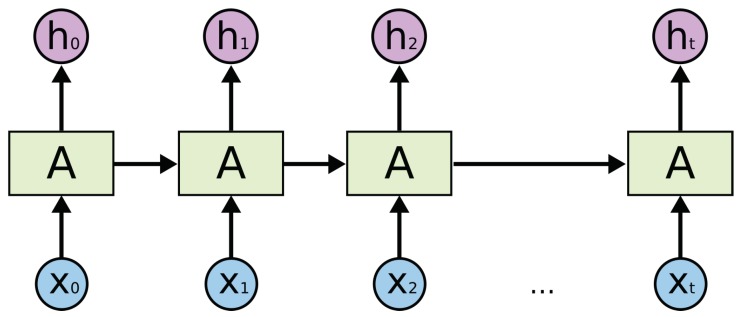
Activity sequence and recurrent neural network (RNN) model.

As depicted in Figure 6, these are spread LSTM networks. $X_{0}-X_{t}$ in this study represent the activity log, and $h_{0}-h_{t}$ represents the prediction result, which is the next activity. We can see that, when the time stamp is *t*, the input of the model is the current activity $X_{t}$ and the past knowledge remembered from t−1 to t−n. It means the model can predict the next by using not only the current activity but also several past activities. This just accords with our assumption that an activity does not happen randomly and that the motivation of the next activity is what the human has done currently. In this way, the prediction accuracy of LSTM is higher than that of the classical machine learning approach; as in the case of the former, more knowledge is considered to model the activity habits of inhabitants.

In addition, we also apply the method in the second stage to the process of prediction. Besides modeling the activity habit, we also utilize LSTM to model the object–usage habit. Therefore, the next object that might be used by the inhabitant can also be predicted using LSTM. Then, we find out the relevant activities associated with the object. Finally, we find the intersection of two prediction results to further improve the prediction performance. By knowing the current activity and the current objects in use, we can predict the next activity with a relatively high accuracy.

## 4. Experiment and Evaluation

In this section, we show the performance of the proposed three-stage framework in recognizing and predicting the activity of the inhabitant. As we have unwrapped the HAR task into three stages, we test performance of all the three stages, respectively. To evaluate the activity recognition performance, we conducted an experiment on an open source dataset. The dataset generated by Ordonez [27] includes 10 ADLs performed by the inhabitant on a daily basis in their own house for 21 days. We selected this dataset because most sensors in this dataset can be replaced by RFID tags to represent the usage in a similar manner.

### 4.1. First Stage

In the first stage, the task is to record the activities that have been performed. There are two key factors that considerably govern the effectiveness of this stage. The first factor is whether the object usage can be detected correctly, and the second one is whether the activity can be labeled properly.

We attach two RFID tags to two commonly used objects: chair and toothbrush. Subsequently, we ask the volunteer to perform a specified interaction with the objects 50 times, respectively, and note the tag states and the corresponding object–usage states. Because both “interfered” and “still” represent no object usage, we treat them as one interaction. In Table 2, we set the following:

- TP represents that the usage is correctly detected;
- TN represents that the interference is correctly detected;
- FP represents that the interference is detected as a usage by mistake;
- FN represents that the usage is detected as an interference by mistake.

Subsequently, the average object usage detection accuracy can be calculated as 97.5%. Moreover, the precision and recall are 96.1% and 99%, respectively. The usage–detection performance is sufficiently good to prove that RFID tags can be used to detect the object usage.

In the experiment, we first find out the objects that are the exclusive representatives for each activity. We then label the data with the activity ID by using those special objects. If the labeled time interval between the start time and the end time has an intersection with the ground truth label, we see it as an appropriate match. The activities that have representative objects can be recognized properly as long as the object usage is detected correctly. However, we find that very few activities can be recognized in this manner, as most of the activities do not have exclusive representative objects. Therefore, we fail to evaluate this part in the first stage, so we have to move to the second stage of our framework to recognize all kinds of activities.

### 4.2. Second Stage

In the second stage, we also utilize the Ordonez dataset to verify our method. The dataset is used for training and testing, respectively.

In the training part, we first classify the object–usage data according to their corresponding activity ID, and each activity contains several representative objects. Subsequently, we calculate the weight of objects in an activity to generate the weight matrix. In the testing part, we use the proposed approach to generate the activity log. After that, we compare the activity log with the ground truth activity log. If the label in ground truth log matches the recognized log, we take a count of TP for this labeled activity ID. However, if the ground truth activity $a_{p}$ is recognized as another activity $a_{q}$, we take a count of $F_{p,q}$.

Here, for easy understanding, we use the verification matrix to represent the recognition result in this stage, as presented in Table 3. As shown in the table, FN is the sum of the row elements apart from the TP in that row, and FP is the sum of the column elements apart from the TP in that column. FN represents the false negative to the activity, and FP represents the false positive to the activity. Then, the precision and recall can be calculated by Equations (7) and (8):

(7) $$precision=\frac{1}{m}\sum_{j=1}^{m}\frac{TP_{j}}{TP_{j}+FP_{j}},$$

(8) $$recall=\frac{1}{m}\sum_{j=1}^{m}\frac{TP_{j}}{TP_{j}+FN_{j}}.$$

The confusion matrix of the recognized activities is presented by Table 4. The activity IDs 1–10 represent 10 activities: leaving, toileting, showering, sleeping, breakfast, dinner, lunch, snack, spare time/TV, and grooming. From the confusion matrix, we can see that the activities having representative objects, for example, activities such as toileting and showering, can be recognized accurately.

According to the calculation, the average precision of our framework in the second stage is 85.0%, and the average recall is 87.9%. In the experiment, we find that the primary element reasonable for false recognition is the temporal-sensitive activities. From Table 4, we can see that activities No. 6 and No. 7 are misidentified as each other three times, respectively. They represent “lunch” and “dinner”, and they are the representative temporal-sensitive activities. In the dataset, activities “breakfast”, “lunch” and “dinner” are treated as three different activities. Although they are different in the temporal space, the representative objects associated with these activities are similar. However, our proposed framework does not consider the temporal knowledge, thereby making it difficult to distinguish those activities. We believe that it is not fair to divide such temporal-sensitive activities merely according to the time because the inhabitants have different daily schedules—for example, if we set the lunch time as from 11:00 a.m. to 1:00 p.m. In addition, the inhabitant may just get up at 11:00 a.m. and have the meal. If we only judge according to the time, it should be “lunch”. However, “breakfast” means the first meal of the day that causes a conflict. Therefore, to recognize such temporal-sensitive activities, we would like to combine the related activities in our future work.

We checked the original activity data, and found that “leaving” is recorded before the inhabitant leaves the house. This means that, when the inhabitant starts to prepare to leave, the interactions are treated as related to “leaving”. In addition, “leaving” is often performed after “grooming”. The segmentation between “leaving” and “grooming” is not noted precisely. Some objects are therefore mistaken as representative of “leaving”. This leads to the fact that “leaving” is confused with “grooming”, and it could be solved by adjusting the definition of activities.

### 4.3. Third Stage

In the process of prediction, we first need to adjust and clean the original data. We treat the same activity that repeats in a short time as a pseudo record and merge them together. Furthermore, we delete some false records that do not meet the common sense. Then, we normalize the data to use it to train the LSTM model. Unlike the second stage, in the third stage, the initial 70% data are utilized for training and the remaining 30% data are for testing.

In the experiment, we build a typical LSTM model on TensorFlow-GPU with Keras as the high-level API. The training epoch is set to 10,000 to ensure the model is well trained. The LSTM model contains four layers: one input layer, two hidden layers and one output layer. The loss is set as ascategoricalcrossentropy, and the optimizer as adam. The timestep and neurons in the hidden layers are hyperparameters. We adjust the hyper parameters to ensure optimal performance of the model.

We find that the test accuracy reaches its optimal value when timestep equals 3. This means that the LSTM model can utilize the past three activities to predict the next activity and achieve the highest accuracy, which accords with our assumption. Furthermore, the accuracy begins to decrease after that, meaning that the pattern of activities can not be too large; otherwise, too much noise will be used.

**Figure 7:**
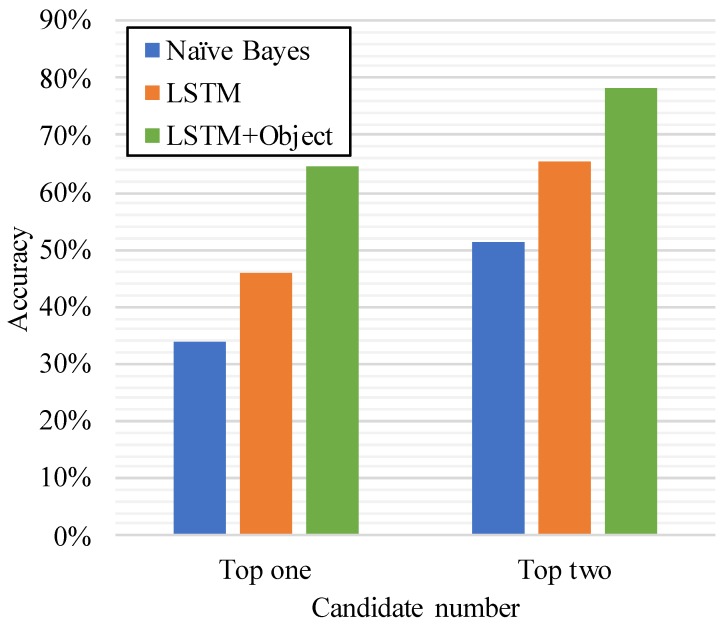
Accuracy of Naive Bayes and long short-term memory (LSTM) solution.

We also compare our model with the classical Naive Bayes method, as depicted in Figure 7. Because the Naive Bayes method only uses the current one activity to predict the next activity, we can see that our solution achieves much higher accuracy than that of Naive Bayes. The top two prediction accuracy reaches 65.2%. Moreover, when we apply the method to the process of prediction in the second stage, the accuracy will be as high is 78.3%.

## 5. Related Work

To enable automatic control in smart homes, a lot of research has been performed in different ways. There are two main kinds of HAR approaches that aim to recognize human activity at home. One is wearable devices, and the other is distributed sensors. Recently, several works have also combined both of these together to build a composite approach. Note that recognition using video cameras has been achieved considerably in HAR [28]. However, it is not appropriates to implement such a vision-based approach because of the privacy problems.

### 5.1. HAR by Wearable Devices

The posture of the human body has a close relationship with the corresponding activity. Because we could not use a camera to monitor the posture of the inhabitant at home, wearable devices become the best choice for doing so. In recent years, deep learning techniques have made good progress to recognize the human posture by using the data produced by wearable devices [29,30]. Represented by Ordonez’s work [30], they put inertial measurement units on the human body to collect the three-dimensional acceleration data and then transform the data to posture by using deep learning algorithms such as Convolutional Neural Network (CNN) and recurrent neural network (RNN). Particularly, more and more smart watches and smartphones have invaded people’s lives with the miniaturization of electronic components. Ronao et al. [31] proposed a way to perform HAR by using smartphone sensor data and CNN, achieving an accuracy of 94.79% with raw sensor data and 95.75% with additional information. Although the size of smart wearable devices is getting smaller and smaller, they still bring inconvenience to a human’s daily life, especially to the elderly and disabled. However, such low-level gesture recognition is unable to provide sufficient knowledge to realize automatic control in smart homes.

### 5.2. HAR by Sensor Networks

Another kind of state-of-the-art approach is to utilize distributing sensors in the home environment to recognize the activity of inhabitants. Researchers have created a variety of binary state-change sensors and attached them to objects that might be used in daily life [32,33]. Using these objects, the sensors may be able to conjecture the activity. The good thing is that such approaches can recognize fine-grained activities, provided they could assign the binary sensors to all the objects at home. However, the weakness of sensors is because of their size and high price. Generally, the home environment contains many tiny objects, which are difficult to attach to binary sensors. Furthermore, the sensors are powered by micro batteries, thereby requiring the replacement of the battery irregularly. These shortages of sensors make the performance of HAR by sensor networks limited to the laboratory only. However, if the sensors could be small and sufficiently cheap, HAR via object usage will be the ideal solution in future smart homes.

## 6. Discussion and Conclusions

In this study, we presented RF-ARP, which is a three-stage framework to deal with the issue of HAR in smart homes. According to the object–usage, our framework can infer the high-level activities and further predict the next possible activity. Without any requirement to the inhabitant, the proposed framework can be widely promoted to a different house at a relative low cost and using less energy. The framework is evaluated on an open source dataset of ADL. The recognition precision can reach 85.0% and the prediction accuracy is 78.3% in the condition of two inhabitants. Compared with the existing work, our framework performs better.

However, there are still some limitations to our current framework. The framework finds it difficult to handle the activities having temporal semantic meaning. This is because our activity recognition method is mainly based on the spatial knowledge. In the future, we would like to move forward a single step to lead the temporal knowledge into our framework. Moreover, we would also like to implement our RF-ARP framework in a living environment to further verify its ability to work in the situation of different and multiple inhabitants, as the dataset used in this study is based on a single-user environment only. In addition, a standard activity definition library needs to be built to reduce the trouble of “cold start”.

References

## Figures and Tables

**Table 1:** Object-usage detection via interaction.

Usage	Tag State	Interaction	Objects
**1**	Covered	Sitting, lying, blocking	Chair, bed, sofa, switch, etc.
	Picked up	Picking up	Knife, toothbrush, chopsticks, etc.
**0**	Interfered	Passing by	All
	Still	Absence	All

**Table 2:** Result of object–usage detection.

Objects	TP	TN	FP	FN
**Chair**	50	49	1	0
**Toothbrush**	49	47	3	1

**Table 3:** Example of verification matrices of TP, FN and FP. TP represents the quantity of true positive; FN represents the quantity of false negative; and FP represents the quantity of false positive.

Activity ID	1	2	3	FN
**1**	$TP_{1}$	$F_{1,2}$	$F_{1,3}$	$FN_{1}$
**2**	$F_{2,1}$	$TP_{2}$	$F_{2,3}$	$FN_{2}$
**3**	$F_{3,1}$	$F_{3,2}$	$TP_{3}$	$FN_{3}$
**FP**	$FP_{1}$	$FP_{2}$	$FP_{3}$	-

**Table 4:** Confusion matrix of recognized activities.

Activity ID	1	2	3	4	5	6	7	8	9	10
**1**	37	0	0	0	0	0	0	0	0	2
**2**	0	91	0	0	0	0	0	0	0	0
**3**	0	0	11	0	0	0	0	0	0	0
**4**	0	0	0	28	0	0	0	0	0	0
**5**	0	0	0	0	22	0	0	0	1	0
**6**	0	0	0	0	0	11	3	0	0	0
**7**	0	0	0	0	0	3	13	1	0	0
**8**	0	0	0	0	0	1	1	45	0	0
**9**	1	0	0	0	0	0	0	2	98	1
**10**	2	0	0	0	0	0	0	0	0	92
